# Polyclonal Endemicity of Carbapenemase-Producing *Klebsiella pneumoniae* in ICUs of a Greek Tertiary Care Hospital

**DOI:** 10.3390/antibiotics11020149

**Published:** 2022-01-25

**Authors:** Efthymia Protonotariou, Georgios Meletis, Dimitrios Pilalas, Paraskevi Mantzana, Areti Tychala, Charalampos Kotzamanidis, Dimitra Papadopoulou, Theofilos Papadopoulos, Michalis Polemis, Simeon Metallidis, Lemonia Skoura

**Affiliations:** 1 Department of Microbiology, AHEPA University Hospital, School of Medicine, Aristotle University of Thessaloniki, S. Kiriakidi str. 1, 54636 Thessaloniki, Greece; protonotariou@auth.gr (E.P.); vimantzana@gmail.com (P.M.); aretich@gmail.com (A.T.); dimitrapapadopoulou8566@gmail.com (D.P.); mollyskoura@gmail.com (L.S.); 2 First Propedeutic Department of Internal Medicine, AHEPA University Hospital, School of Medicine, Aristotle University of Thessaloniki, 54636 Thessaloniki, Greece; pilalas_jim@hotmail.com; 3 Hellenic Agricultural Organisation-DIMITRA, Veterinary Research Institute of Thessaloniki, Campus of Thermi, 57001 Thermi, Greece; kotzam@vri.gr; 4 Department of Microbiology and Infectious Diseases, Faculty of Veterinary Medicine, School of Health Sciences, Aristotle University of Thessaloniki, 54636 Thessaloniki, Greece; theofilos23@vet.auth.gr; 5 Central Public Health Laboratory, National Public Health Organization, 16672 Vari, Greece; m.polemis@eody.gov.gr; 6 First Department of Internal Medicine, Infectious Diseases Division, AHEPA University Hospital, School of Medicine, Aristotle University of Thessaloniki, 54636 Thessaloniki, Greece; symeonam@auth.gr

**Keywords:** *Klebsiella pneumoniae*, carbapenemases, NDM, KPC, VIM, OXA-48, molecular epidemiology, PFGE

## Abstract

Carbapenemase-producing *Klebsiella pneumoniae* (CPKP) emerged in Greece in 2002 and became endemic thereafter. Driven by a notable variability in the phenotypic testing results for carbapenemase production in *K. pneumoniae* isolates from the intensive care units (ICUs) of our hospital, we performed a study to assess the molecular epidemiology of CPKP isolated between 2016 and 2019 using pulse-field gel electrophoresis (PFGE) including isolates recovered from 165 single patients. We investigated the molecular relatedness among strains recovered from rectal surveillance cultures and from respective subsequent infections due to CPKP in the same individual (48/165 cases). For the optimal interpretation of our findings, we carried out a systematic review regarding the clonality of CPKP isolated from clinical samples in ICUs in Europe. In our study, we identified 128 distinguishable pulsotypes and 17 clusters that indicated extended dissemination of CPKP within the hospital ICU setting throughout the study period. Among the clinical isolates, 122 harbored KPC genes (74%), 2 harbored KPC+NDM (1.2%), 38 harbored NDM (23%), 1 harbored NDM+OXA-48 (0.6%), 1 harbored NDM+VIM (0.6%) and 1 harbored the VIM (0.6%) gene. Multiple CPKP strains in our hospital have achieved sustained transmission. The polyclonal endemicity of CPKP presents a further threat for the selection of pathogens resistant to last-resort antimicrobial agents.

## 1. Introduction

In recent years, hospital-acquired infections caused by carbapenem-resistant Gram negative bacteria, especially carbapenem-resistant *Klebsiella pneumoniae* (CRKP), have been observed worldwide causing important public health problems and posing serious infection control issues. CRKP are opportunistic pathogens that cause infections with high morbidity and mortality mainly in hospitalized patients [1,2]. In Europe, the burden of CRKP predominantly affects the south and the east. According to the annual report of the European Centre for Disease Prevention and Control on antimicrobial resistance in Europe, 66.3% of the reported invasive *K. pneumoniae* isolates in Greece during 2020 were resistant to carbapenems. 

Relatively high carbapenem resistance rates were also observed in Romania (48%), Italy (29%) and Bulgaria (28%), while, in the majority of the EU countries, this proportion was below 10% [3]. High carbapenem resistance trends were also observed in other non-EU neighboring countries: Bosnia, Herzegovina, Georgia, the Russian Federation, Serbia and Turkey reported proportions between 25% and 50% whereas, Belarus, the Republic of Moldova and the Ukraine reported proportions exceeding 50% [4].

The most common mechanism of carbapenem resistance among Enterobacterales and, thus, *K. pneumoniae* is the production of carbapenemases. Carbapenemases are β-lactamases able to hydrolyze all β-lactams, including carbapenems, and are categorized into several types. The carbapenemases most commonly encountered in Greece belong in three classes: class A *K. pneumoniae* carbapenemase (KPC), class B Verona imipenemase (VIM) and New Delhi metallo-β-lactamase (NDM) and class D oxacillinase-48 (OXA-48) [5]. 

Carbapenemase-producing *K. pneumoniae* (CPKP) emerged in Greece in 2002; they were of VIM-type, were involved in various outbreaks and soon became endemic in many hospitals all over the country. VIM-type carbapenemase-producers often belonged to different clones with ST147 being the predominant multi-locus sequence type (MLST) [6]. In 2007, KPC-producing *K. pneumoniae* isolates were introduced in Greek hospitals and rapidly dominated [7]. 

Greek KPC-CPKP mostly belonged to the worldwide successful hyperepidemic clone ST258, often associated with multi-drug resistant (MDR) phenotype [8]. The emergence of NDM in CPKP strains in Greece took place in 2011; the majority of them belonged to ST11 and were involved in oligoclonal outbreaks or sporadic cases [9]. OXA-48 type carbapenemases are the most prevalent class D enzymes identified in CPKP strains. The first OXA-48 was detected in Athens, Greece in 2012 and belonged to ST11 [10]. 

Carbapenemase-encoding genes spread fast via horizontal gene transfer together with other resistance determinants within the *K. pneumoniae* species in hospital settings, thus, dramatically restricting the available treatment options [11]. Moreover, the local epidemiology and the limited availability for isolation of affected patients in separate rooms in Greek hospitals undermine the efforts for effective infection control strategies.

*K. pneumoniae* is characterized by a high variety of antimicrobial resistance genes as well as a wide ecological distribution. Thus, in addition to its significance as a nosocomial pathogen (especially the hypervirulent phenotype), *K. pneumoniae* is considered as one of the most important bacterial species contributing in the dissemination of antimicrobial resistance genes to other human pathogens [12].

*K. pneumoniae* has the ability to colonize various mucosal surfaces, including the upper respiratory and the gastrointestinal gut. Among hospitalized patients, colonization rates in the nasopharynx are up to 19%, while it can reach as high as 77% in the gastrointestinal tract. Gut colonization often precedes and serves as a reservoir for transmission to other body sites resulting in the development of subsequent infections [13]. The duration of gut colonization with multi-drug resistant (MDR) bacteria, such as carbapenem-resistant *K. pneumoniae* varies from 43 to 387 days [14].

Driven by a notable variability in the phenotypic testing results for carbapenemase production and the types of carbapenemases present in *K. pneumoniae* isolates in our hospital, we performed a study to assess the molecular epidemiology of CPKP isolated between 2016 and 2019. Additionally, we investigated the molecular relatedness among strains recovered by rectal surveillance cultures and by respective subsequent infections due to CPKP in the same individual. In order to put our findings in context, we also performed a systematic review regarding the clonality of CPKP isolated from clinical samples originating in intensive care units (ICUs) in Europe.

## 2. Results

### 2.1. Carbapenemase Detection

During the study period (January 2016–June 2019) 165 single-patient clinical CRKP were analyzed; the isolates were recovered from 115 patients hospitalized in ICU 1, 6 patients in ICU 2 and 44 patients in ICU 3. Clinical samples included blood (*n* = 36), central venous catheters (*n* = 27), bronchial secretions (*n* = 51), urine (*n* = 20), pus (*n* = 13), wound swabs (*n* = 12), pleural fluid (*n* = 1), peritoneal fluid (*n* = 2), nasal swab (*n* = 1) and cerebrospinal fluid (*n* = 2).

Phenotypic and molecular testing revealed that all CRKP isolates harbored at least one carbapenemase often combined with ESBL activity. Among the clinical isolates, 122 harbored the KPC gene (73.95%), 2 KPC + NDM (1.21%), 38 NDM (23.04%), 1 NDM + OXA-48 (0.60%), 1 NDM + VIM (0.60%) and 1 VIM (0.60%).

### 2.2. Pulse-Field Gel Electrophoresis

**Figure 1:**
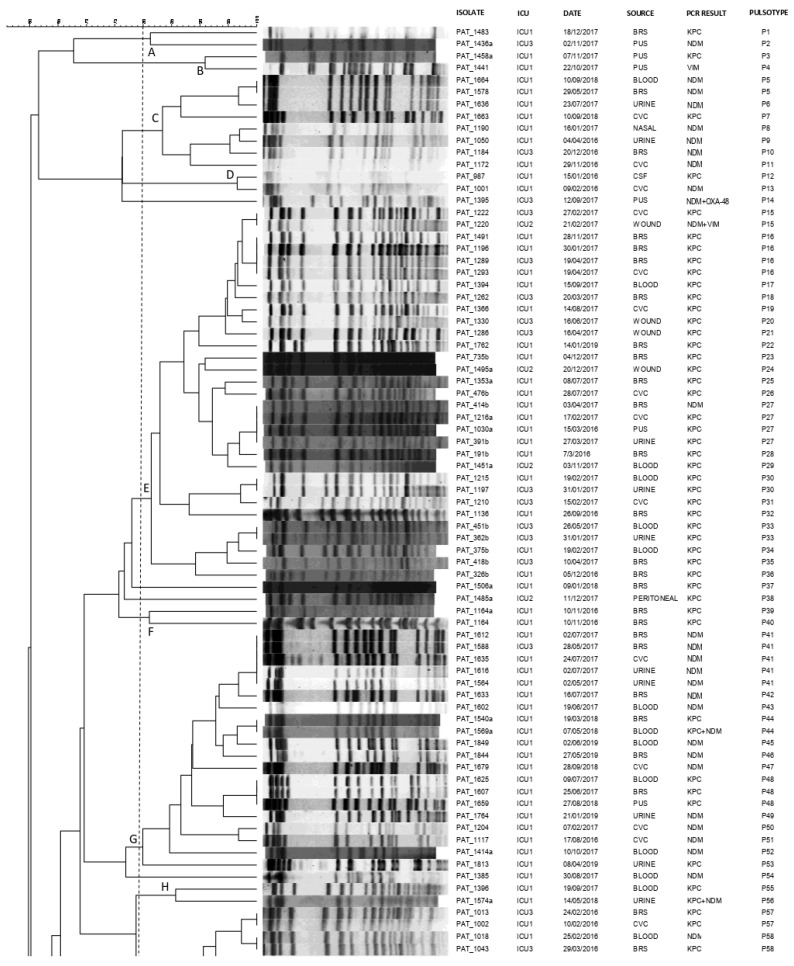

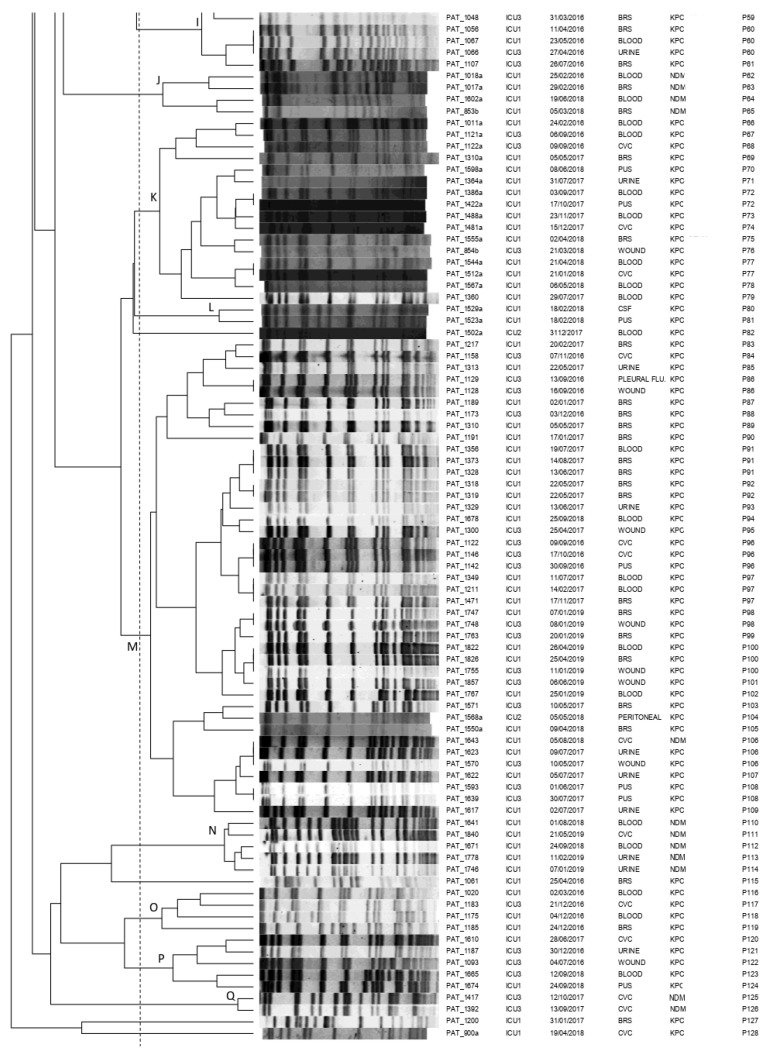
Pulsotypes of the 165 clinical carbapenemase-producing *K. pneumoniae* isolates. Clusters (**A**–**Q**) were defined at a similarity level of 80%.PAT: patient; ICU: intensive care unit; CVC: central venous catheter; BRS: bronchial secretions; CSF: cerebrospinal fluid; P: pulsotype.

All the 165 clinical CRKP isolates were typeable by pulse-field gel electrophoresis (PFGE) following digestion by restriction enzyme XbaI, revealing 128 distinguishable pulsotypes (P1-P128; Figure 1).

At a similarity level of 80% or above, the majority of CPKP isolates (95.6%, 158/165) were assigned into 17 clusters (A-Q), demonstrating multiclonal dissemination. The remaining seven genomes resulted to be unrelated and were consequently classified as sporadic isolates. KPC as well as NDM genetic determinants demonstrated polyclonal dissemination being present in 14 and 11 distinct clones, respectively. 

In more detail, four predominant clusters E, G, K, and M consisting of 31, 19, 16 and 41 CRKP isolates, respectively, were identified: isolates of cluster G were almost exclusively obtained from ICU 1 (18 of 19), while clusters E, K, M consisted of clinical isolates from all ICUs under study. Interestingly, within the above clusters, indistinguishable pulsotypes shared by isolates from different patients and different ICUs were identified. Furthermore, looking at indistinguishable pulsotypes, we could identify common pulsotypes among isolates obtained from different patients during different time periods of the study (P5, P27, P48 and P106).

**Figure 2:**
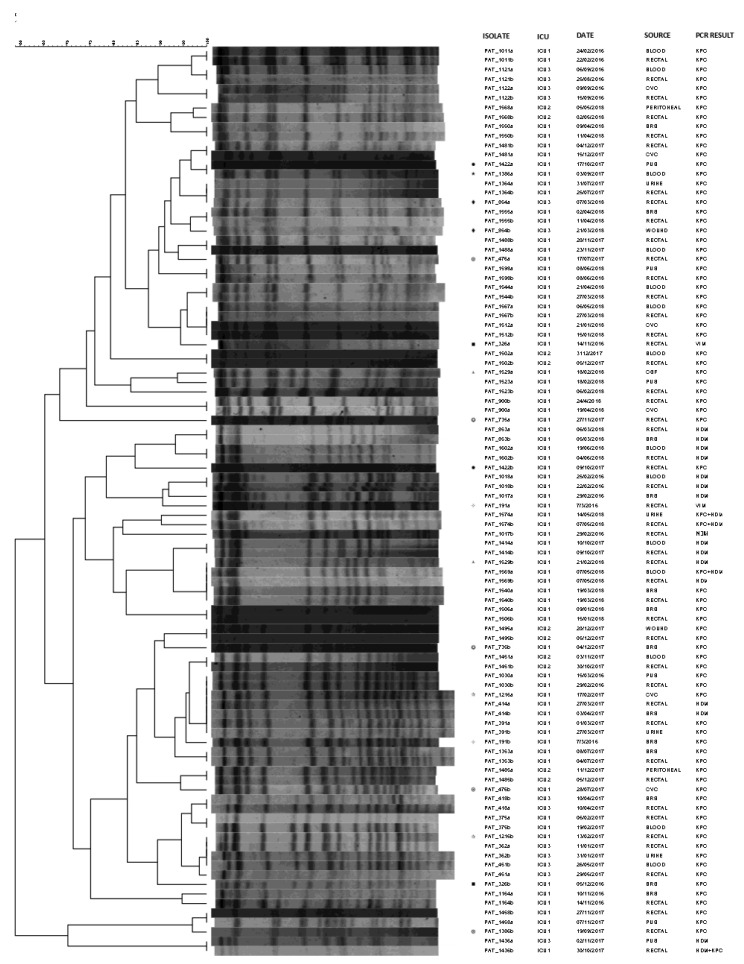
Pulsotypes of the 48 rectal-clinical carbapenemase-producing *K. pneumoniae* isolate pairs. Pairs with different pulsotypes are marked with the same symbol. PAT: patient; ICU: intensive care unit; CVC: central venous catheter; BRS: bronchial secretions; CSF: cerebrospinal fluid.

We also used PFGE analysis for revealing the genetic association among CRKP strains from rectal and clinical samples of 48 representative patients. According to PFGE, in the majority of the cases (81.3%, 39 of 48), the clinical and rectal strains from the same patients were identical (Figure 2). Different pulsotypes were observed for pairs PAT_1422a/b, PAT_1386a/b, PAT_854a/b, PAT_476a/b, PAT_326a/b, PAT_1529a/b, PAT_735a/b, PAT_191a/b and PAT_1216a/b.

Six clinical-rectal surveillance pairs presented different PCR results (PAT_1017a/b, PAT_1436a/b, PAT_1529a/b, PAT_1569a/b, PAT_191a/b and PAT_326a/b). Among them, PAT_1017a/b, PAT_1529a/b, PAT_191a/b and PAT_326a/b showed also different PFGE profiles. Overall, different pulsotypes were observed for pairs PAT_1422a/b, PAT_1386a/b, PAT_854a/b, PAT_476a/b, PAT_1529a/b, PAT_735a/b, PAT_191a/b and PAT_1216a/b even though the PCR results were identical among the clinical and the rectal samples.

### 2.3. Non-Susceptibility Rates of CRKP Isolates in the Hospital

**Figure 3:**
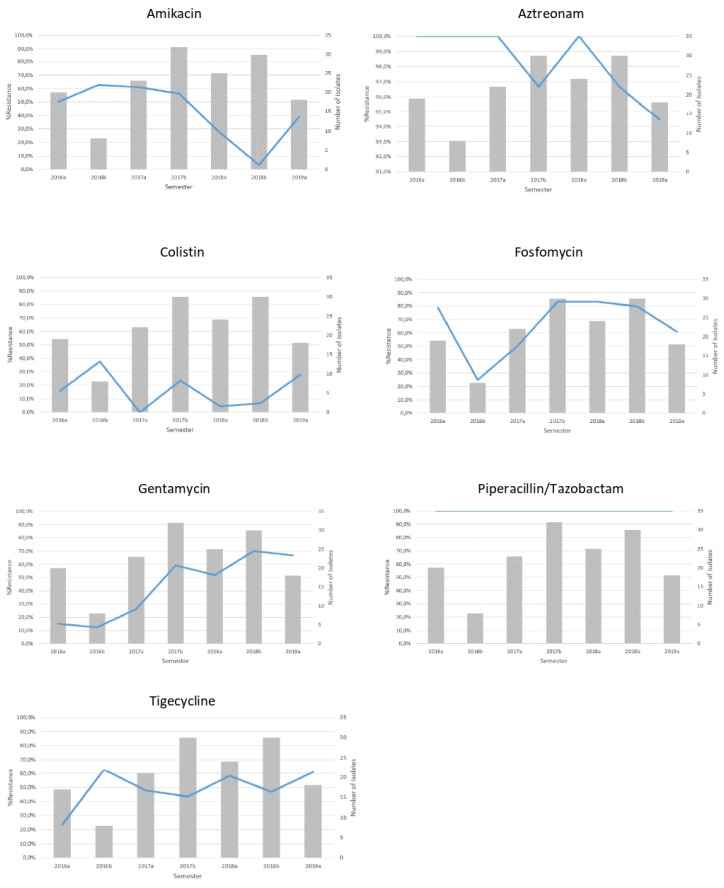
Non-susceptibility rates (line) and number (bars) of single patient imipenem non-susceptible *K. pneumoniae* isolates recovered from the hospital’s ICUs per semester.

The non-susceptibility rates of CRKP isolates in our institution’s ICUs for amikacin, aztreonam, colistin, fosfomycin, gentamicin, piperacillin/tazobactam and tigecycline in isolates during the study period are shown in Table 1 and Figure 3. CRKP isolates presented with high level of resistance to both meropenem and imipenem (MIC_50_ ≥ 16 mg/L) throughout the study years. On the other hand, a significant increase in resistance was observed for gentamycin ranging from 15% in the first semester of 2016 to 66.7% in the first semester of 2019. In regard to tigecycline, an increase was also observed (23.5% in the 2016a semester to 61.1% in the 2019a semester). As for colistin, an increase was observed (15.8% in the 2016a semester to 27.8% in the 2019a semester).

### 2.4. Systematic Review Results

Our systematic review search strategy yielded 290 results. After implementation of the exclusion criteria, 39 studies remained. Fourteen studies reported monoclonal dissemination. Data extracted from the remaining 25 studies are reported in Table 2.

## 3. Discussion

The present study evaluated the type of carbapenemases and the molecular epidemiology of *K. pneumoniae* strains circulating in the ICUs of a tertiary hospital in Thessaloniki, Greece between 2016 and 2019. During the study years, KPC was the predominant carbapenemase; NDM were also present, and a few double-carbapenemase-producers were isolated. On the basis of PFGE, a total of 17 different CRKP transmission clusters were identified.

CRKP isolates have been introduced in our hospital since 2004. At that time, no phenotypic or molecular testing was performed to reveal the type of carbapenemase up to 2010. From 2010 to 2014, phenotypic testing among CRKP isolates revealed that the majority carried KPC enzymes (60%), 25% produced MBL, and 7% co-produced KPC and MBL enzymes. In the time period of 2011–2014, we also observed the first OXA-48 producers in our hospital at a rate of 1.7%. Molecular testing showed that KPC positive strains harbored the *bla*_KPC-2_, while MBL-positive strains harbored *bla*_VIM-1_. 

During 2013–2015, an oligoclonal outbreak caused by 45 CRKP occurred in our hospital [40]. All the patients were hospitalized in the three intensive care units of the hospital, and 17 (68%) of them developed bloodstream infections; the overall mortality of the patients involved in the outbreak was 48% (12/25). Molecular testing verified that all 45 *K. pneumoniae* isolates co-harbored *bla*_KPC-2_ and *bla*_VIM-1_ and were associated with OmpK35 deficiency and OmpK36 porin loss. PFGE clustered all isolates into a single clonal type, and multi-locus sequence typing (MLST) assigned them to the emerging high-risk ST147 clonal lineage. 

Starting from 2014 until 2016, while KPC producers still prevailed (approximately 75% of the CRKP), we observed a shift among MBL producers from *bla*_VIM-1_ towards *bla*_NDM-1_. More specifically, VIM-type carbapenemases decreased from 17.6% in 2014 to 6.7% in 2016, whereas NDM-type increased from 1.2% in 2014 to 19.4% in 2016 [41]. In the years 2013–2015, the co-production of KPC and MBL enzymes corresponded to approximately 10% of all CRKP strains [35]. Finally, in 2019, we isolated a strain carrying both NDM-1 and OXA-48 genes classified as ST-11 [42].

In our study, PFGE analysis identified 128 distinguishable pulsotypes and 17 clusters indicating an extended dissemination of CRKP within the hospital setting. Moreover, the dissemination took place over a long-time frame since we included in our study isolates recovered during a 3.5-year period. The presence of identical isolates in all three ICUs highlights their successful dissemination through different hospital wards. More worryingly, the persistence of certain strains throughout the whole study period, despite the various infection control measures that were applied, reflects the difficulties that undermine the efforts for their eradication once they are well-established in a certain geographical area. Indeed, such strains may have persisted in the hospital or/and may have been re-introduced by carrier admissions.

In fact, most of the CRKP carriers that later presented a CRKP infection had identical pulsotypes between their rectal and clinical isolates. The most probable explanation for this finding is that gut colonization preceded infection. In some cases, however, the pulsotypes of rectal and clinical isolates were different indicating that the infection was caused by another nosocomial *K. pneumoniae*. 

There were also two clinical-rectal pairs (PAT_1436a/b and PAT_1569a/b) that harbored different carbapenemase-encoding genes according to PCR results but had identical PFGE pulsotypes. This could be explained by the mobilization of mobile genetic elements, most likely by the loss and acquisition of plasmids [43] even though an infection by a different isolate of the same pulsotype harboring different resistance determinants could not be excluded. PFGE studies in our hospital performed in several CRKP strains during the period of 2011 to 2013 revealed that KPC strains prevailed and that the majority of them belonged to two distinct clones (unpublished data). 

A similar pattern of carbapenemases was observed in Hippokration General Hospital of Thessaloniki, Greece where KPC carbapenemases have prevailed among CRKP since 2009 outnumbering the VIM-type carbapenemases that predominated previously [44]. In the same report, KPC-producers belonged to two distinct clones, the predominant of which correspond to the hyperepidemic Greek clone. In a multicenter nationwide surveillance study conducted in several Greek hospitals for CRKP from 2014 to 2016, NDM-producing isolates belonged mainly to one clone, whereas KPC, VIM, OXA-48 and double carbapenemase-producers were mainly categorized in three clones [45]. On the contrary, in our study, both KPC and NDM CRKP isolates showed extremely multiclonal profiles.

In our study, we also observed a rise in the resistance rates of tigecycline, gentamicin and colistin. This is in accordance with other studies reporting elevated resistance rates to last resort antibiotics driven, among other factors, by a vicious cycle of increased last resort antibiotic consumption and subsequent resistance [46].

Our systematic review results showed that non-monoclonal dissemination of *K. pneumoniae* strains in ICU settings has been described before in countries participating in the EARS-Net (Table 2). Of note, such observations have been reported almost exclusively by Mediterranean countries, mainly from Greece and Italy, and this is in accordance with the epidemiological situation of the region regarding carbapenem-resistance determinants. However, and despite the heterogeneity of settings and methods used, most studies reported rather oligoclonal transmission with further identification of sporadic cases. 

In our study, multiple clones circulating simultaneously achieved sustainable dissemination and, according to our knowledge and our systematic review results, this is the first time that such polyclonal dissemination has been observed in Europe. This multi-clonal PFGE observation highlights a possible additional reason for their endemic persistence in our hospital even though infection control measures, including hand hygiene, surveillance for colonization among high-risk patients and contact precautions have been established.

In this context, active surveillance with rectal swab cultures is of outmost importance to control the spread of these pathogens by isolation or cohorting of the colonized patients [29]. However, the spread of CRKP in an endemic environmental niche is a dynamic and multifaceted phenomenon that involves many variables. In a similar situation, more than one CRKP clone may be simultaneously present in the hospital; whereas new admissions may be CRKP carriers most likely by previous hospitalizations in the same or other hospitals. Consequently, a multi-clonal spread is very likely to occur and, even when a previously colonized subject presents a CRKP infection, it is not certain that this infection is directly related to the strain that colonized the patient upon admission.

Our study has several limitations. A multi-centric study would be able to evaluate whether the epidemiological pattern that we observed in our single center study was an isolated phenomenon or more widespread. Including non-ICU along with ICU *K. pneumoniae* strains would yield a more complete picture for their dissemination. In our analysis, we did not include clinical patient level information, and this limits our ability to draw conclusions with regard to precipitating factors. Finally, we were not able to employ sequencing-based methods to better characterize the molecular epidemiology of the strains included in our study.

## 4. Materials and Methods

### 4.1. Study Design

This was a retrospective study that was carried out at AHEPA University Hospital, a 700-bed institution with three ICUs, a central surgical and medical ICU (8 beds, ICU 1), a surgical ICU (4 beds, ICU 2) and a cardiosurgical ICU (5 beds, ICU 3) as well as surgical and internal medicine departments. The study was approved by the institutional medical scientific board. Sample related patient data were retrieved from the laboratory database.

CRKP clinical isolates, recovered in the aforementioned ICUs between January 2016 and June 2019 from 165 single patients, were included in the study. In 48 cases, a rectal isolate (isolated upon admission in ICU for infection control purposes) and a subsequent clinical isolate (isolated by an infection that occurred during ICU stay) were considered, thus forming 48 pairs of surveillance-clinical isolates. Isolates taken from the remaining 117 patients were all recovered from clinical specimens only.

Rectal swabs taken from ICU patients upon admission were inoculated on MacConkey agar plates supplemented with meropenem and ceftazidime discs. All Gram-negative colonies that grew after 24 h of incubation near the discs were further identified and *K. pneumoniae* isolates were tested for carbapenemase production with phenotypic and molecular techniques. For clinical specimens, standard laboratory procedures were followed depending on each specimen source.

Bacterial identification and antimicrobial susceptibility testing were performed with the Vitek2 automated system (Biomerieux, Marcy-l’Étoile, France). Furthermore, the minimum inhibitory concentration of tigecycline was determined by E-test (Liofilchem, Roseto degli Abruzzi, Italy) and for colistin using the broth microdilution method (Liofilchem, Roseto degli Abruzzi, Italy). The results of all antimicrobial testing were interpreted in accordance with the CLSI criteria. For tigecycline, the breakpoints recommended by the United States Food and Drug Administration were used (susceptible: MIC ≤ 2 mg/L; resistant: MIC ≥ 8 mg/L).

### 4.2. Carbapenemase Detection

All isolates were phenotypically screened for carbapenemase production with the Modified Hodge Test (MHT), [47] while the type of MBL or KPC was assessed with the Combined Disk Test (CDT) [48]. Following phenotypic identification, PCR assays were performed for carbapenemase-encoding genes using specific primers for *bla*_KPC_, *bla*_VIM_, *bla*_IMP_, *bla*_NDM_ and *bla*_OXA-48_ (Appendix A) [49].

### 4.3. Pulse-Field Gel Electrophoresis

The genetic relationship among the CPKP isolates was determined by PFGE according to standardized protocol [50] with the XbaI endonuclease (New England Biolabs, Beverly, MA, USA) by using a CHEF-DR III apparatus (Bio-Rad Laboratories Inc., Hercules, CA, USA) for the separation of DNA fragments. XbaI-digested DNA from *Salmonella enterica* serotype Braenderup H9812 was used as a reference size standard, while PFGE patterns were digitally analyzed using the FPQuest (Bio-Rad Laboratories Pty Ltd., Hercules, CA, USA) software package. 

PFGE profiles were compared using the Dice correlation coefficient with a maximum position tolerance of 1.5% and an optimization of 1.5%. Similarity clustering analysis was performed by using the Unweighted Pair Group Method using Averages (UPGMA), and a dendrogram was generated. Two PFGE profiles were classified as indistinguishable if the DNA fragment patterns matched each other completely, while clusters were selected using a cutoff at the 80% level of genetic similarity.

### 4.4. Non-Susceptibility Rates of CRKP Isolates

For every semester of the study period, we determined the *K. pneumoniae* non-susceptibility rates of amikacin, aztreonam, colistin, fosfomycin, gentamicin, piperacillin/tazobactam and tigecycline in imipenem non-susceptible single patient isolates from the ICUs using the CLSI 2020 breakpoints.

### 4.5. Systematic Review

In order to evaluate the extent of non-monoclonal transmission of CRKP strains in the intensive care environment in countries participating in the European Antimicrobial Resistance Surveillance Network (EARS-Net), we undertook a systematic review of the recent literature. We searched MEDLINE via PubMed from 1 January 2000 to 28 April 2021, implementing the search strategy described in Appendix B. Titles and abstracts were screened for studies, which incorporated a molecular epidemiology investigation (PFGE or sequencing methods) of CRKP strains, including samples obtained from ICU patients. 

We excluded reviews, case reports, studies restricted to environmental samples, studies that did not explicitly include any ICU clinical samples and studies not conducted in EARS-Net participating countries. No language and patient age restrictions were applied. Eligibility assessment was conducted in duplicate (D.P. and G.M.) and discrepancies were adjudicated by a third author (E.P.). 

Next, studies reporting monoclonal transmission were excluded, and, from the remaining studies, we extracted the following data: study setting and eligible samples, sample type (clinical versus surveillance and infection versus colonization), the number of carbapenem-resistant isolates and mechanism of resistance detected, the method used to investigate molecular epidemiology and the number and size of clusters involving ICU patients. If ICU-level data were not available, hospital-wide data were reported. Data extraction was conducted by A.T., G.M., T.P., D.P. and E.P. All steps were conducted in duplicate.

## 5. Conclusions

In our study, we demonstrated that CPKP in our hospital belonged to a great variety of pulsotypes and clusters, thus, indicating their extended dissemination within the hospital ICU settings. Among them, KPC carbapenemase predominated. The presence of multiple clones harboring variable resistance-determinants poses additional challenges. Further studies are required to identify suitable infection control strategies in a setting of polyclonal dissemination within a context of carbapenem resistance endemicity. Our results highlight the need to urgently reinforce infection-control measures along with antimicrobial stewardship and together with a generous increase in the nosocomial budget in order to contain the transmission of antibiotic resistant organisms.

## Figures and Tables

**Table 1:** Imipenem resistant *K. pneumoniae* susceptibility rates per semester among single patient isolates recovered from the hospital’s ICUs during the study period.

Semester	Antimicrobial	No Tested	R	I	S	R%	I%	S%
2016a	Amikacin	20	10	1	9	50%	5%	45%
	Aztreonam	19	19	0	0	100%	0%	0%
	Gentamicin	20	3	0	17	15%	0%	85%
	Piperacillin/Tazobactam	20	20	0	0	100%	0%	0%
	Colistin	19	3	0	16	15.8%	0%	84.2%
	Tigecycline	17	4	13	0	23.5%	76.5%	0%
	Fosfomycin	19	15	0	4	79.0%	0%	21.0%
2016b	Amikacin	8	5	1	2	62.5%	12.5%	25%
	Aztreonam	8	8	0	0	100%	0%	0%
	Gentamicin	8	1	0	7	12.5%	0%	87.5%
	Piperacillin/Tazobactam	8	8	0	0	100%	0%	0%
	Colistin	8	3	0	5	37.5%	0%	62.5%
	Tigecycline	8	5	2	1	62.5%	25%	12.5%
	Fosfomycin	8	2	0	6	25%	0%	75%
2017a	Amikacin	23	14	0	9	60.9%	0%	39.1%
	Aztreonam	22	22	0	0	100%	0%	0%
	Gentamicin	23	6	1	16	26.1%	4.3%	69.6%
	Piperacillin/Tazobactam	23	23	0	0	100%	0%	0%
	Colistin	22	0	0	22	0%	0%	100%
	Tigecycline	21	10	5	6	47.6%	23.8%	28.6%
	Fosfomycin	22	11	0	11	50%	0%	50%
2017b	Amikacin	32	18	3	11	56.2%	9.4%	34.4%
	Aztreonam	30	29	0	1	96.7%	0%	3.3%
	Gentamicin	32	19	3	10	59.4%	9.4%	31.2%
	Piperacillin/Tazobactam	32	32	0	0	100%	0%	0%
	Colistin	30	7	0	23	23.3%	0%	76.7%
	Tigecycline	30	13	12	5	43.3%	40%	16.7%
	Fosfomycin	30	25	0	5	83.3%	0%	16.7%
2018a	Amikacin	25	7	4	14	28%	16%	56%
	Aztreonam	24	24	0	0	100%	0%	0%
	Gentamicin	25	13	0	12	52%	0%	48%
	Piperacillin/Tazobactam	25	25	0	0	100%	0%	0%
	Colistin	24	1	0	23	4.2%	0%	95.8%
	Tigecycline	24	14	8	2	58.3%	33.3%	8.4%
	Fosfomycin	24	20	0	4	83.3%	0%	16.7%
2018b	Amikacin	30	1	3	26	3.3%	10%	86.7%
	Aztreonam	30	29	0	1	96.7%	0%	3.3%
	Gentamicin	30	21	0	9	70%	0%	30%
	Piperacillin/Tazobactam	30	30	0	0	100%	0%	0%
	Colistin	30	2	0	28	6.7%	0%	93.3%
	Tigecycline	30	14	14	2	46.7%	46.7%	6.6%
	Fosfomycin	30	24	0	6	80%	0%	20%
2019a	Amikacin	18	7	1	10	38.9%	5.5%	55.6%
	Aztreonam	18	17	0	1	94.4%	0%	5.6%
	Gentamicin	18	12	0	6	66.7%	0%	33.3%
	Piperacillin/Tazobactam	18	18	0	0	100%	0%	0%
	Colistin	18	5	0	13	27.8%	0%	72.2%
	Tigecycline	18	11	5	2	61.1%	27.8%	11.1%
	Fosfomycin	18	11	0	7	61.1%	0%	38.9%

**Table 2:** Studies reporting non-monoclonal dissemination of carbapenem-resistant *Klebsiella pneumoniae* clinical strains, including ICU populations.

Study	Setting	Time Period	Study Population (Eligible)	Sample Type (Clinical vs. Surveillance, Infection vs. Colonization)	Number Of CR-Isolates and Mechanism of Resistance	Method(s)	Number of Clusters and Isolates/Cluster
(Hernández-García et al., 2021) [15]	11 Portuguese hospitals	June 2017 to July 2018	Colistin-susceptible and -resistant MDR *Escherichia* spp. (*n* = 30) and *Klebsiella* spp. (*n* = 78) isolates	Lower respiratory, intra-abdominal and urinary tract infections of ICU patients	31 CRKP 3 CR *E. coli* KPC-3 (*n* = 14) was the most common carbapenemase followed by OXA-48 (*n* = 3) and OXA-181 (*n* = 3)	WGS	A great diversity of *Kp* high-risk clones was observed associated with the KPC-3 carbapenemase, including some lineages first reported in Portuguese Hospitals (ST13, ST34, ST405, ST1563, ST4331)
(Fontana et al., 2020) [16]	Tor Vergata University Hospital, Rome, Italy	May 2013 to Dec 2016	147 consecutive, non-replicate clinical strains of CRE from different wards	Blood cultures	*bla*_KPC_ was present in 121/147 (87%) strains, mainly *Kp*. The remaining strains carried *bla*_VIM_ or *bla*_OXA-48_	WGS MLST	5 clusters with 2 to 9 strains
(Galani et al., 2020) [17]	2 ICUs of Hygeia General Hospital, Athens, Greece	Sept to Oct 2019	7 patients colonized or infected with ceftazidime-avibactam (CZA)-resistant *K. pneumoniae*	Colonization or infection	co-produced KPC-2 and the novel plasmid-borne VEB-25	WGS MLST PFGE	PFGE classified the isolates in 2 pulsotypes however, all but one, belonged to the second pulsotype
(Ferrari et al., 2019) [18]	1 cardiorespiratory ICU with 8 beds in a 900-bed Hospital in Pavia, Italy	Aug 2015 to May 2016	23 patients with 32 CRKP isolates were analyzed	12 colonized 11 infected	(9.4% carried KPC-2 and 90.6% KPC-3; All 32 analyzed isolates carried at least one ESBL gene (3.1% CTX-M-15, 3.1% SHV-1, 87.5% SHV-11, 6.3% SHV-12	WGS	Multi-clone epidemic event - 26 of the 32 isolates belong to three genome clusters and the remaining six were classified as sporadic - The first genome cluster was composed of MDR ST512 - The second infection cluster comprised four other genomes of ST512 - The third cluster ST258 colonized 12 patients
(Mavroidi et al., 2020) [19]	Kostantinopouleio-Patission G. Hospital, Athens, Greece 280-bed general hospital (including a nine-bed ICU)	Jan 2014 to Dec 2016	248 CRKP in ICU	Bronchial secretions (*n* = 105), blood (*n* = 53), central venous catheters (*n* = 39), urine (*n* = 28)	The majority of CRKP from BSIs were OXA-48 producers (*n* = 23) and KPC producers (*n* = 18) whereas the remaining 12 isolates produced and/or MBLs (6 VIM, 3 OXA-48+VIM, and 3 NDM producers)	MLST	ST101 (OXA-48) ST258 (KPC) ST11 (NDM)
(Gona et al., 2019) [20]	1 teaching hospital in Catania, Italy	Oct 2016 to Jan 2018	Neonatal ICU. All confirmed CRKP isolates included	12 infections, 1 colonization	13 isolates all NDM+OXA-48	PFGE MLST Core genome MLST	1 pulsotype Clinical isolates included a common MLST (ST101), and 2 novel STs (ST3366 and ST3367), which differ from ST101 by a single nucleotide of rpoB gene. The cgMLST method accurately characterized transmission events of the 13 *K. pneumoniae* isolates in three clusters: A containing only ST101, B containing only ST3367, and C containing both ST3366 and ST101 due to the close relationship between ST101 and ST3366. Four isolates were included in cluster A, two isolates in cluster B, and seven isolates in cluster C.
(Karampatakis et al., 2018) [21]	Hippokration General Hospital, Thessaloniki, Greece 900 beds	Aug 2012 to Nov 2014	Conducted in a 9-bed polyvalent ICU. 143 CRKP selected randomly	Infection or colonization	44 CRKP (mostly KPC and VIM, 2 NDM, 2 OXA-48, 1 NDM+OXA-48, 1 KPC+OXA-48)	PFGE	10 pulsotypes A: 24 isolates (all KPC) A2: 1 KPC B: 11all VIM (2 VIM+KPC) C: 2 D: 1 E: 1 F: 1 G: 1 H: 1 I: 1 No relevant further information available
(Papadimitriou-Olivgeris et al., 2018) [22]	University Hospital of Patras, Greece 800 beds.	2010–2016 Months not specified	Isolates from hospitalized patients in the ICU. It was a matched 1:2 case–control study conducted among critically ill patients in order to identify the risk factors of ColR-Kp and TigR-Kp bacteraemia	Blood infections	110 included in PFGE 91 KPC, 4 VIM, 5 KPC+VIM, 10 NDM	PFGE	3 pulsotypes A: 76 mostly KPC B: 24 mostly KPC C: 10 only NDM
(Avgoulea et al., 2018) [23]	Tzaneio Hospital, Athens, Greece 450 beds	June 2014	ICU patients; The aim of the study was to analyze the mode of spread and the characteristics of epidemic OXA-48-Kp strains responsible for bloodstream infections in ICU patients emerged in June 2014	Blood infections	19 selected OXA-48	PFGE MLST	2 pulsotypes 2 STs Pulsotype A was ST147 (the first 4 cases-PDR) Pulsotype B was ST101 (the next cases-MDR)
(Ripabelli et al., 2018) [24]	Antonio Cardarelli Hospital, Molise, Italy	2010, 2014–2016 Months not specified	30 from the ICU 10 from wards	Infection (*n* = 27) or colonization (*n* = 13)	23 WILD TYPE (2010), 17 NON-WILD TYPE (KPC) (2014–2016)	PFGE RAPD	16 clusters and 26 pulsotypes 23 clusters and 33 patterns 2010 and 2014-16 isolates were grouped in different clusters by both methods
(Bartolini et al., 2017) [25]	Padova Hospital, Italy	1/2015–9/2106	Adult patients from the ICU, surgical and medical department and patients with epidemiological link to persons with CPKP isolates	Rectal swabs and clinical samples	311 CPKP: 258 KPC, 17 OXA-48, 12 NDM	MLST	16 different CPKP strains without predominance: 35 ST-258, 85 ST-512, 32 ST-745, 54 ST-307, 22 ST-554, 5 ST-15, 11 ST-16, 3 ST-101, 3 ST-11, 1 ST-37, 1 ST-45, 1 ST-211, 1 ST-398, 1 ST-147, 1 ST-1458
(Mavroidi et al., 2016) [26]	Kostantinopouleio-Patission G. Hospital, Athens, Greece 280-beds	July 2012 to Dec 2013	Imipenem and meropenem resistant isolates of all hospital’s department	Surveillance rectal swabs and clinical samples	135 CPKP. 19 were colistin resistant and all of them harbored the *bla*_KPC_ gene.	MLST	The 19 COL-R CP-Kp isolates belonged to 2 STs: 18 to ST-258 and 1 ST-383 lineages.
(Bonura et al., 2015) [27]	3 acute general hospitals in Palermo, Italy	March–Aug 2014	All carbapenem resistant isolates of all hospital’s department	Isolates from any sight of infection or colonisation	94 carbapenem non susceptible isolates all KPC-3 producers	PFGE and MLST	10 pulsotypes: A(4), B(1), C(subtypes:C1(15), C2(2)), D(subtypes: D1(22),D2(3),D3(1),D4(1)), E(1), F(1), G(4), H(1), I(1), O(37). 10STs. 37 ST258, 1 ST512, 27 ST307, 17 ST273, 4 ST405, 4 ST101, 1 ST15, 1 ST147, 1 ST323, 1 ST491
(Onori et al., 2015) [28]	Ospedale di Circolo e Fondazione Macchi Varese, Italy	Jan 2011 to March 2013	Infections due to carbapenem-resistant *Kp*	Clinical samples	16 CRKP isolates. 3 harbored the *bla*_KPC-2_ and 13 the *bla*_KPC-3_ variant.	WGS	2 STs. 10 isolates belonged to ST512 and 6 to ST258.
(Parisi et al., 2015) [29]	Padova Hospital, Italy	Jan 2012 to Dec 2014	Patients from the Intensive care, surgery and medical departments	Clinical and surveillance samples	496 CPKP strains out of which 436 tested with molecular methods: 432 KPC, 3 OXA-48, 1 NDM	MLST	MLST available for 238/496 isolates. In total 15 STs were identified: 90 ST258, 86 ST512, 31 ST745, 5 ST15, 2 ST101, 1 ST868, 6 ST307, 3 ST554, 1 ST392, 1 ST437, 1 ST1207, 1 ST1326, 1 ST395, 1 ST1199, 1 ST1543.
(Katsiari et al., 2015) [30]	Konstantopouleio General Hospital, Athens, Greece	2010–2012	279-bed tertiary-care hospital. Athens. 1 ICU, 9 beds, all imipenem-resistant *Kp*	clinical or surveillance	6 CRKP isolates (48 KPC-producers and 13 VIM-producers) were recovered from 58 ICU patients.	PFGE Representative isolates to MLST	Seven types (A–G) according to 85% similarity, 42 (69%) to A cluster. -MLST type ST258 Type A was further divided into 12 subtypes (A1–A12) according to 100% pattern similarity, 10/13 VIM classified in type B
(Mezzatesta et al., 2014) [31]	1 general ICU Catania Hospital, Italy	1–31 July 2013	ICU *Kp* isolates responsible for severe infections	clinical isolates	25 *Kp* 57 patients, all harbored *bla*_KPC-3_.	PFGE MLST	4 pulsotypes among all the KPC-producing *Kp* (A, B, C and D), MLST 4 distinct STs: All pulsotype A strains belonged to ST258 and pulsotype B was categorized as ST512 detected in most isolates. Pulsotypes C and D were also identified, in a few strains, as ST147 and ST395, respectively.
(Papadimitriou-Olivgeris et al., 2014) [32]	General ICU (13 beds) of the University Hospital of Patras, Greece	26 months	Hospital of Patras, Greece, a 770-bed teaching hospital.	Recovered from clinical or rectal samples from patients (*n* = 273) who stayed more than 6 days in the ICU	53 KPC-*Kp* isolates from 48 patients All 53 KPC-*Kp* isolates carried the *bla_KPC_*	PFGE	Two PFGE types (A and B) were identified, with 36 (67.9%) strains belonging to PFGE type A and 17 (32.1%) to PFGE type B.
(Capone et al., 2013) [33]	9 hospitals of Rome, Italy	Dec 2010 to May 2011	1 teaching institution, 6 tertiary hospitals, 1 clinical and research institute, and 1 long-term care facility, with a total of 4000 beds, ranging from 100 to 1200 beds per centre	97 patients *Kp* strain showing reduced susceptibility to ertapenem (MIC 1 mg/L), Clinical samples urine (*n* = 34), blood (*n* = 34), lower respiratory tract (*n* = 13), surgical wound (*n* = 8), intraabdominal fluid (*n* = 7), CVC tips (*n* = 12), rectal swab (*n* = 3) and cerebrospinal fluid (*n* = 1)	Strains producing *bla*_KPC-3_ were identified in 89 patients, *bla*_VIM_ in three patients and *bla*_CTX-M-15_ plus porin defects in the remaining five patients. 1 isolate per patient	MLST	Among strains producing KPC-3, two major clones identified by MLST: ST512 and ST258, KPC-3 was also identified in clones ST646 (new ST), ST650 (new ST), ST14 and ST101. The *bla*_VIM-1_ gene was identified in clones ST646, ST647 and ST648 (three new STs). Among strains producing ESBL combined with outer membrane protein (OmpK) defects, three belonged to ST37, and the other was assigned to the new ST649
(Tofteland et al., 2013) [34]	A 12-bed mixed ICU in the Arendal hospital, Norway	Nov 2007 to April 2011	KPC-producing outbreak strains	Clinical and surveillance samples/Infection or colonization	7 KPC-2 strains from 7 patients	PFGE MLST	A 6 ST258 B 1 ST461
(Mammina et al., 2012) [35]	24 beds in two general ICUs, in 1 acute general hospital in Palermo, Italy	June to Dec 2011	All colistin-resistant *Kp* isolates during this period (possible outbreak) irrespective of their source patient and clinical sample	58 colistin-resistant *Kp* isolates were recovered from 28 patients irrespective of their source	52 isolates carried the *bla*_KPC-3_ and SHV-11. 6 isolates susceptible to carbapenems, resistant to fluoroquinolones and aminoglycosides	Rep-PCR	Al 52 isolates carried the *bla*_KPC-3_ gene belonging in sequence type ST258 Rep-PCR confirmed that the colistin-resistant isolates belonged to three different clusters, one that contained all ST258 KPC-3 producing isolates, and two clusters with unrelated patterns including the ST15 and ST273isolates
(Richter et al., 2012) [36]	2 hospitals (1580 and 300 beds) in Padua, Italy	June 2009 to Dec 2011	Phenotypic and genotypic investigation for KPC in clinical samples	Infection or colonization	189 KPC-2 or KPC-3 strains	PFGE MLST ERIC	4 PFGE profiles ST37, ST147,ST258, ST307, ST437, ST510,ST512, ST527, ST554 3 ERIC profiles
(Sánchez-Romero et al., 2012) [37]	613 bed teaching hospital, Madrid, Spain -52 ICU beds	Jan to Dec 2009	Any carbapenem non susceptible strain from ICU patients	Clinical or surveillance/ Infection or colonization	55 patients harbouring VIM-1 strains/molecular epidemiology for 99 strains	PFGE MLST	-PFGE: A 54, B 4 -MLST: 6 A isolates ST15, 3 B isolates ST340
(Souli et al., 2010) [38]	University General Hospital Attikon 635-bed teaching hospital, Athens, Greece—1 ICU (18 beds till 10/2008, 21 after)	Jan 2007 to Dec 2008	Any clinical *Kp* isolate with imipenem or meropenem MIC > 1 mg/mL producing KPC (hospital-wide)	Clinical or surveillance samples/Infection or colonization	50 KPC-2 isolates (34 ICU/16 non- ICU, 18 infections (9 ICU, 9 non- ICU)/32 colonization)	PFGE	4 PFGE types: A 41, B 6, C 1, D 2 Only A was responsible for infections
(Giakkoupi et al., 2003) [39]	3 teaching hospitals in Athens, Greece	Sep to Dec 2002	ICU patients with archived imipenem non susceptible specimens	Clinical samples/ at least 12 infections	17 *bla*_VIM-1_ strains from 17 patients	PFGE	4 PFGE types: the majority (5 and 10 isolates) belonged to two types

MLST: Multi-Locus Sequence Typing; WGS: Whole Genome Sequencing; ERIC: enterobacterial repetitive intergenic consensus; MDR: multi-drug resistant; PDR: pan-drug resistant; *Kp*: *Klebsiella* pneumoniae; ColR-*Kp*: Colistin resistant-*Kp*; TigR-*Kp*: Tigecycline resistant-*Kp*.

## Data Availability

Not applicable.

## Appendix A. Primers for Carbapenemase Detection

**Gene**	**Primers (5′–3′)**
*bla*_KPC_	TGTCACTGTATCGCCGTC
	TATTTTTCCGAGATGGGTGAC
*bla*_IMP_	CTACCGCAGCAGAGTCTTTG
	AACCAGTTTTGCCTTACCAT
*bla*_VIM_	TCTACATGACCGCGTCTGTC
	TGTGCTTTGACAACGTTCGC
*bla*_NDM-1_	GGTTTGGCGATCTGGTTTTC
	CGGAATGGCTCATCACGATC
*bla*_OXA-48_	TTGGTGGCATCGATTATCGG
	GAGCACTTCTTTTGTGATGGC

## Appendix B. Systematic Review Search Strategy

#1 (critical care OR ICU OR intensive care OR critical ill OR critical illness OR critically ill OR “Intensive Care Units”[Mesh] OR “Critical Care”[Mesh] OR “Critical Illness”[Mesh])
#2 carbapenem* OR meropenem OR imipenem OR ertapenem
#3 Klebsiella
#4 (epidem* OR outbreak OR clon* OR strain*)
#5 (PFGE OR puls* OR genom* OR typing OR sequenc* OR MLST OR NGS OR WGS OR cgMLST or wgMLST OR MLVA)
#6 (“1 January 2000”[Date–Publication]: “28 April 2021”[Date–Publication])

Final search(29 April 2021): #1 AND #2 AND #3 AND #4 AND #5 AND #6 → 290 results
